# Genetic variation in *TIMP1 *but not *MMPs *predict excess FEV_1 _decline in two general population-based cohorts

**DOI:** 10.1186/1465-9921-12-57

**Published:** 2011-04-27

**Authors:** CC van Diemen, DS Postma, M Siedlinski, A Blokstra, HA Smit, HM Boezen

**Affiliations:** 1 Departments of Epidemiology, University Medical Center Groningen, University of Groningen, Groningen, The Netherlands; 2 Department of Pulmonology, University Medical Center Groningen, University of Groningen, Groningen, The Netherlands; 3 National Institute of Public Health and the Environment (RIVM), Bilthoven, The Netherlands; 4 Julius Center, University of Utrecht, The Netherlands

## Abstract

**Background:**

An imbalance in Matrix MetalloProteases (MMPs) and Tissue Inhibitors of MMPs (TIMPs) contributes to Chronic Obstructive Pulmonary Disease (COPD) development. Longitudinal studies investigating Single Nucleotide Polymorphisms (SNPs) in *MMPs *and *TIMPs *with respect to COPD development and lung function decline in the general population are lacking.

**Methods:**

We genotyped SNPs in *MMP1 *(G-1607GG), *MMP2 *(-1306 C/T), *MMP9 *(3 tagging SNPs), *MMP12 *(A-82G and Asn357Ser) and *TIMP1 *(Phe124Phe and Ile158Ile) in 1390 Caucasians with multiple FEV_1 _measurements from a prospective cohort study in the general population. FEV_1 _decline was analyzed using linear mixed effect models adjusted for confounders. Analyses of the X-chromosomal *TIMP1 *gene were stratified according to sex. All significant associations were repeated in an independent general population cohort (n = 1152).

**Results:**

*MMP2 *-1306 TT genotype carriers had excess FEV_1 _decline (-4.0 ml/yr, p = 0.03) compared to wild type carriers. *TIMP1 *Ile158Ile predicted significant excess FEV_1 _decline in both males and females. *TIMP1 *Phe124Phe predicted significant excess FEV_1 _decline in males only, which was replicated (p = 0.10) in the second cohort. The *MMP2 *and *TIMP1 *Ile158Ile associations were not replicated. Although power was limited, we did not find associations with COPD development.

**Conclusions:**

We for the first time show that *TIMP1 *Phe124Phe contributes to excess FEV_1 _decline in two independent prospective cohorts, albeit not quite reaching conventional statistical significance in the replication cohort. SNPs in *MMPs *evidently do not contribute to FEV_1 _decline in the general population.

## Background

Chronic Obstructive Pulmonary Disease (COPD) is characterized by chronic airway inflammation, associated with extracellular matrix (ECM) degradation and loss of elastic recoil of lung tissue. The *Matrix Metalloprotease *(*MMP*) gene family is thought to participate in the excessive collagenolytic and elastolytic activity that contributes to ECM destruction. MMPs are a family of secreted and membrane associated zinc-dependent endopeptidases, capable of cleaving ECM and non-matrix proteins. Many studies have shown that MMP1, MMP2, MMP9, MMP12 protein and mRNA levels are higher in lung tissue and induced sputum of COPD patients than of controls [1-6].

Proteolytic activities of the MMPs are normally tightly controlled in several ways, e.g. by transcriptional regulation, activation of latent zymogen and interaction with endogenous inhibitors of MMPs, the Tissue Inhibitors of MMPs (TIMPs). Especially the imbalance between MMPs and TIMPs has been proposed to play a major role in ECM destruction and development of COPD, a pulmonary disease strongly associated with smoking. While most COPD patients have smoked, only a subset of smokers develops COPD, and it is likely that the susceptibility to smoking is genetically determined. It is thus reasonable that genetic determinants of the balance between MMPs and TIMPs contribute to COPD development.

Single nucleotide polymorphisms (SNPs) have been described in the promoter regions of *MMP1, MMP2, MMP9 *and *MMP12 *and they can alter their expression levels [7-10]. Joos *et al*. showed that SNPs in the *MMP1 *and *MMP12 *promoter regions are more prevalent in subjects with fast FEV_1 _decline compared to subjects with no FEV_1 _decline in a cohort of current smokers with mild to moderate airway obstruction [11]. SNPs in MMP12 have been variably associated with lung function, i.e. with higher lung function in children with asthma and adult smokers and additionally with a reduced risk of COPD in adult smokers [12] and increased risk of severe COPD [13]. A *MMP9 *promoter SNP has been associated with emphysema in a case-control study in a Japanese population [13]. and with COPD in a Chinese population [14]. In contrast, the promoter SNP in *MMP2*, a biologically plausible candidate for COPD, as well as *TIMP1 *and *TIMP2 *SNPs have not been studied in relation to COPD development or FEV_1 _decline. Whereas *TIMP2 *does not contain SNPs known to alter function or expression, two synonymous *TIMP1 *SNPs in the gene region responsible for binding and inactivating of MMP9 have been associated with asthma [15]. Thus, given the role of TIMP proteins in inhibiting effects of metalloproteases SNPs in these genes can conceivably play a role in COPD development.

Unraveling the genetics of *MMPs *and *TIMPs *in COPD development may identify subjects who may specifically benefit from novel treatments like synthetic MMP inhibitors that effectively prevent smoke-induced COPD in animal models. Therefore, we studied SNPs in *MMP1, MMP2, MMP9, MMP12*, and *TIMP1 *and their interaction in relation to accelerated FEV_1 _decline and COPD development in a general population cohort. To verify our findings, we investigated whether significant associations could be replicated in an independent cohort of the general population.

## Methods

### Subjects

We genotyped DNA from 1390 subjects of the Vlagtwedde/Vlaardingen cohort that participated in the last survey in 1989/1990 [16]. This general population-based cohort of Caucasians of Dutch descent started in 1965 and surveys were performed at three year intervals. At each survey, lung function measurements were performed using standardized protocols and questionnaires were completed (see additional file 1). The selection of the cohort and details of the study have been described previously [16]. The study protocol was approved by the local university hospital's medical ethics committee and participants gave written informed consent.

As a replication cohort we used data from a random sample of 1152 subjects from the Doetinchem cohort, which is part of the larger MORGEN study [17,18]. The MORGEN study was a random sample of the general population of the Netherlands. Participants of the Doetinchem study underwent spirometry in 1994-1997 and 5 years later in 1999-2003. Characteristics of both study populations are presented in table 1. We identified subjects with COPD using the GOLD criteria (GOLD stage II or higher, i.e. FEV_1_/VC< 70% and FEV_1_<80% predicted) [19].

**Table 1:** Characteristics of the Vlagtwedde/Vlaardingen and Doetinchem cohorts

	Vlagtwedde/Vlaardingen N = 1390	Doetinchem N = 1152
Age at last survey, yrs	52 (35-79)	50 (31-71)
Males, %	51	47
Pack-years	9.0 (0-262.1)	5 (0-84)
Never smokers, n (%)	445 (32.0)	371 (32.2)
last FEV_1_%pred*	93.5 (36.0-138.1)	106.6 (39.1-150.7)
ΔFEV_1_, ml/yr^†^	-21.1 (-121;155)	-28.7 (-292; 130)
FEV_1 _values, n	7 (1-8)	2 (2-2)
Yrs of follow-up	21 (0-25)	5 (5-5)
GOLD stage > II,n (%)	186 (13.4)	37 (3.2)

Data are presented as median (range)

* FEV_1 _% predicted at the last surveys of Vlagtwedde/Vlaardingen cohort (1989/1990) and the Doetinchem cohort (1999-2003)

^† ^calculated as last-first FEV_1_/years participated

### DNA collection and genotyping

DNA collection and the genotyping protocol of the Vlagtwedde/Vlaardingen study have been described previously [16]. We genotyped functional SNPs G-1607GG in *MMP1*, C-1306T in *MMP2*, A-82G and Asn357Ser (A/G) in *MMP12*. No tagging SNPs are known for *TIMP1*, therefore we decided to genotype two noncoding SNPs, previously associated with asthma [15]. Phe124Phe (T/C) and Ile158Ile (C/T) in *TIMP1*. In *TIMP2*, we genotyped G-418C. With Haploview, using genotype data from the HapMap project [20,21]we selected 3 haplotype tagging SNPs for *MMP9 *that tag haplotypes with a frequency above 5% in *MMP9 *including 5 kb flanking regions at both the 3'UTR and 5'UTR: rs6065912, rs3918278 and rs8113877. Characteristics of the genotyped SNP are presented in table 2

**Table 2:** Characteristics of the genotyped SNPs

SNP name	rs number	Chromosome position of gene	Functionality
*MMP1 *G-1607GG	rs1799750	11q22-q23	G-insertion generates a new 5'-GGA-3' core recognition sequence for members of the ETS family of transcription factors

*MMP2 *C-1306T	rs243865	16q23	T-allele disrupts a Sp1 binding site, thereby lowering the promoter activity approximately twofold in macrophages and epithelial cells

*MMP9 *rs6065912	rs6065912	20q11.2-13.1	tagging SNP for *MMP9 *gene

*MMP9 *rs3918278	rs3918278		tagging SNP for *MMP9 *gene

*MMP9 *rs8113877	rs8113877		tagging SNP for *MMP9 *gene

*MMP12 *A-82G	rs2276109	11q22.2-22.5	A-allele has higher affinity for transcription factor AP-1 and and higher gene expression in reporter gene assays

*MMP12 *Asn357Ser	rs652438		located in the coding region of the hemopexin domain that is responsible for MMP12 activity, while the function of this polymorphism remains unknown

*TIMP1 *Phe124Phe	rs4898	Xp11.3-11.23	unknown

*TIMP1 *Ile158Ile	rs11551797		unknown

*TIMP2 *G-418C			

Abbreviations: SNP Single Nucleotide Polymorphism; MMP Matrix Metallo Protease; TIMP Tissue Inhibitor of MMP; ETS E26 transformation-specific; Sp1 Stimulating Protein 1; AP-1 Activator Protein-1

The SNPs that were significantly associated with excess FEV_1 _decline or COPD development in the Vlagtwedde/Vlaardingen population were genotyped in the Doetinchem cohort by KBioscience http://www.kbioscience.co.uk using a patent-protected system (KASPar). We used the statistical software R, "genetics" package (version 1.9.1) to determine whether the SNPs were in Hardy Weinberg equilibrium and linkage disequilibrium.

### Statistics

All *TIMP1 *analyses were stratified according to sex, since this gene is located on the X-chromosome. To investigate the effect of SNPs on annual FEV_1 _decline in the Vlagtwedde/Vlaardingen cohort, we used Linear Mixed Effect (LME) models with adjustment for potential confounders (i.e. sex, first FEV_1 _after age 30 years, pack-years) using a general genetic model (see additional file 1) [16,22]. We tested whether there was an interactive effect of *TIMP1 *and *MMP *SNPs on FEV_1 _decline by introducing their interaction term into the model. We used ANOVA and linear regression models to study SNP effects on first and last available FEV_1 _and FEV_1_/VC (adjusted for sex, age, pack-years, and height in regression models). Differences in genotype frequencies of single SNPs for all genes and additionally haplotype frequencies in *MMP9 *between subjects with and without COPD were tested using Chi-square tests.

The SNPs that were significantly associated with excess FEV_1 _decline or COPD development in the Vlagtwedde/Vlaardingen population (p values < 0.05; tested 2-sided) were genotyped in the Doetinchem cohort for verification. FEV_1 _decline in the Doetinchem cohort was calculated based on FEV_1 _decline between the two surveys and genotype effects were tested using linear regression analyses, adjusted for sex, age, pack-years and baseline FEV_1_

Statistical analyses were performed using SPSS (version 14.0.1 for Windows), S-Plus (version 7), the statistical package R (version 1.9.1) [23]. and Chaplin [24,25].

## Results

Allelic frequencies for the minor alleles of the *MMP *and *TIMP *SNPs in the Vlagtwedde/Vlaardingen cohort were comparable to those reported in the NCBI dbSNP database: *MMP1 *G-1607GG 0.51, *MMP2 *C-1306T 0.27, *MMP9 *rs3918278 0.03, *MMP9 *rs6065912 0.12, *MMP9 *rs8113877 0.40, *MMP12 *A-82G 0.15, *MMP12 *Asn357Ser 0.03, *TIMP1 *Ile158Ile in males 0.01, in females 0.01, and *TIMP1 *Phe124Phe in males 0.50, and in females 0.53. All SNPs were in Hardy Weinberg equilibrium. The SNPs in *MMP9 *were in high LD (r^2 ^>0.8).

### Association of MMP SNPs in Vlagtwedde/Vlaardingen

**Figure 1:**
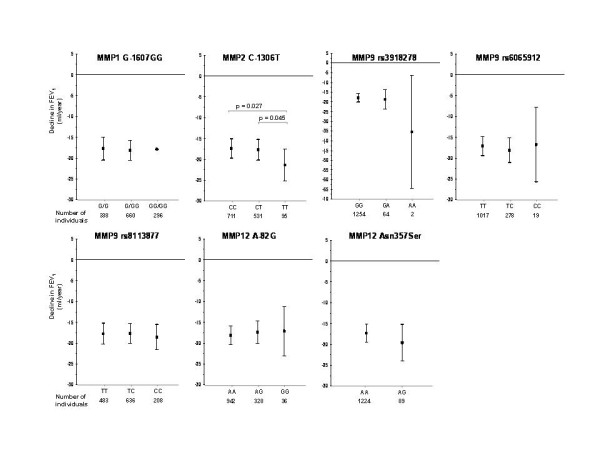
**Effect of SNPs in *MMP *genes on longitudinal decline in FEV_1_**. Mean adjusted declines in FEV_1 _(in ml/yr) are shown per genotype; bars represent 95% confidence intervals.

*MMP2 *C-1306T was significantly associated with accelerated longitudinal decline in FEV_1 _in the total population (TT-genotype -4.0 ml/yr excess decline compared to CC-genotype, p = 0.027, figure 1), and was also associated with a lower mean FEV_1 _% predicted (CC: 92.5, CT: 93.5, TT: 88.5% predicted; p = 0.013) at the last survey. This association remained significant after adjustment for packyears of smoking in linear regression models. SNPs in *MMP1, MMP12 *and SNPs and haplotypes in *MMP9 *were not significantly associated with longitudinal FEV_1 _decline, level of lung function or presence of COPD (GOLD stage ≥ II) (table 3), although power was limited for the latter. Since smoking upregulates MMP activity [26]. we also analyzed FEV_1 _decline with respect to interaction of the SNPs and smoking. These interaction-terms were not significant.

**Table 3:** *MMP *SNPs and development of COPD in Vlagtwedde/Vlaardingen (GOLD stage ≥ II)

SNP		No COPD N (%)	COPD N (%)	P value	SNP		No COPD N (%)	COPD N (%)	P value
*MMP1 *G-1607GG	G	295 (26.4)	44 (24.7)	0.845	*MMP9 *rs6065912	TT	873 (77.0)	145 (80.1)	0.576
	G&GG	565 (50.6)	94 (52.8)			TC	245 (21.6)	18 (33.2)	
	GG	257 (23.0)	40 (22.5)			CC	16 (1.4)	3 (1.7)	
									
*MMP2 *C-1306T	CC	609 (52.8)	103 (55.4)	0.180	*MMP9 *rs8113877	TT	408 (35.8)	76 (40.6)	0.137
	CT	466 (40.5)	65 (34.9)			TC	559 (49.0)	77 (41.2)	
	TT	77 (6.7)	18 (9.7)			CC	174 (15.2)	34 (18.2)	
									
*MMP12 *Asn357Ser	AA	1054 (93.1)	171 (94.0)	0.673	*MMP9 *rs3918278	GG	1078 (94.7)	177 (96.7)	0.122
	AG	78 (6.9)	11 (6.0)			GA	59 (5.2)	5 (2.7)	
	GG	0 (0)	0 (0)			AA	1 (0.1)	1 (0.6)	
									
*MMP12 *A-82G	AA	812 (72.2)	130 (71.4)	0.831					
	AG	281 (25.0)	48 (26.4)						
	GG	32 (2.8)	4 (2.2)						

### Association of TIMP1 SNPs in Vlagtwedde/Vlaardingen

**Figure 2:**
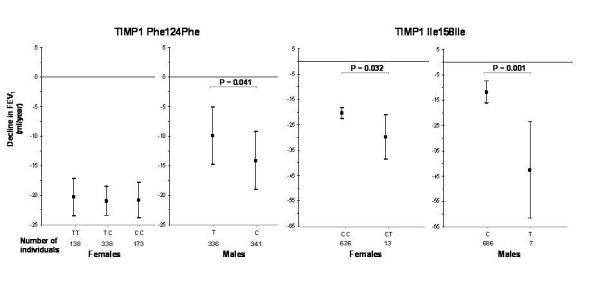
**Effect of SNPs in *TIMP1 *on longitudinal decline in FEV_1_, stratified by sex**. Mean adjusted declines in FEV_1 _(in ml/yr) are shown per genotype; bars represent 95% confidence intervals.

The *TIMP1 *Phe124Phe SNP was associated with excess FEV_1 _decline in males only (-4.2 ml/yr excess FEV_1 _decline compared to wild type (p = 0.041, figure 2). We found that the *TIMP1 *Ile158Ile SNP was associated with excess longitudinal FEV_1 _decline in both males and females (-30.7 ml/yr respectively -9.5 ml/yr excess FEV_1 _decline compared to wild type, p = 0.001 and p = 0.031 respectively, figure 2). The minor allele of the Ile158Ile SNP was more prevalent in females with COPD than without COPD: CT genotype, 6.5% and 1.5% respectively, p = 0.051 (table 4). The *TIMP1 *Phe124Phe SNP was not associated with COPD, although power to detect such an association was low. SNPs in *TIMP1 *were not associated with level of lung function cross-sectionally.

**Table 4:** *TIMP1 *SNPs and development of COPD in Vlagtwedde/Vlaardingen (GOLD stage ≥ II), stratified by sex.

FEMALES					MALES				
		**No COPD N (%)**	**COPD N (%)**	**P value**			**No COPD N (%)**	**COPD N (%)**	**P value**

*TIMP1 *Ile158Ile	CC	572 (98.5)	43 (93.5)	0.051	*TIMP1 *Ile158Ile	C	533 (98.9)	130 (99.2)	0.724
	CT	9 (1.5)	3 (6.5)			T	6 (1.1)	1 (0.8)	
	TT	0 (0)	0 (0)						
									
*TIMP1 *Phe124Phe	TT	122 (21.1)	12 (25.0)	0.298	*TIMP1 *Phe124Phe	T	264 (50.4)	60 (45.8)	0.348
	TC	308 (53.2)	20 (41.7)			C	260 (49.6)	71 (54.2)	
	CC	149 (25.7)	16 (33.3)						

### Interaction of TIMP1 and MMP genes on FEV_1 _decline in Vlagtwedde/Vlaardingen

We found significant associations of *TIMP1 *and *MMP2 *SNPs with FEV_1 _decline. To test for interaction between these genes, we included interaction terms of *TIMP1 *and *MMP2 *SNPs in our models on FEV_1 _decline, and stratified the analyses by sex. These interaction terms were not significant.

### Replication of significant findings in an independent population cohort

To investigate whether results were not found due to chance, we analyzed genes that were significantly associated with FEV_1 _level or decline in the Vlagtwedde/Vlaardingen cohort, i.e. *MMP2 *and *TIMP1*, in an independent cohort of the general population. Genotype frequencies in the Doetinchem population were similar and not statistically different from the Vlagtwedde/Vlaardingen population (table 5). The *TIMP1 *Phe124Phe SNP was associated with excess FEV_1 _decline in males (T allele -7.6 ml/yr compared to wild type, p = 0.10), similarly to the findings in the Vlagtwedde/Vlaardingen cohort, although with lower significance. In contrast to the findings in Vlagtwedde/Vlaardingen, *TIMP1 *Ile158Ile was not associated with excess decline, but with less FEV_1 _decline in females (42.9 ml/yr less decline compared to wild type, p = 0.008), but not in males. The *MMP2 *C-1306T was not significantly associated with excess FEV_1 _decline or lower FEV_1_% predicted in the Doetinchem cohort.

**Table 5:** Genotype distribution of *MMP2 *and *TIMP1 *SNPs in Vlagtwedde/Vlaardingen and Doetinchem.

		Vlagtwedde/Vlaardingen N (%)	Doetinchem N (%)	P value					
					
*MMP2 *C-1306T	CC	734 (53.0)	609 (55.1)	0.055					
	CT	552 (39.9)	443 (40.1)						
	TT	98 (7.1)	53 (4.8)						
		**FEMALES**					**MALES**		

		**Vlagtwedde/Vlaardingen N (%)**	**Doetinchem N (%)**	**P value**			**Vlagtwedde/Vlaardingen N (%)**	**Doetinchem N (%)**	**P value**

*TIMP1 *Ile158Ile	CC	636 (98.0)	602 (99.2)	0.079	*TIMP1 *Ile158Ile	C	686 (99.0)	526 (99.4)	0.394
	CT	13 (2.0)	5 (0.8)			T	7 (1.0)	3 (0.6)	
	TT	0 (0)	0 (0)						
									
*TIMP1 *Phe124Phe	TT	138 (21.3)	135 (23.4)	0.657	*TIMP1 *Phe124Phe	T	336 (49.6)	240 (47.6)	0.494
	TC	338 (52.0)	295 (51.1)			C	341 (50.4)	264 (52.4)	
	CC	173 (26.7)	147 (25.5)						

*TIMP1 *SNP distributions are stratified by sex.

To increase the power of the studies, we additionally tested for association for COPD development and FEV_1_% predicted in pooled analyses of the Vlagtwedde/Vlaardingen and Doetinchem cohorts. We only found a significant association for COPD with *TIMP1 *Ile158Ile in females (OR = 4.3, 95% CI = 1.2-15.3, p = 0.015), similar as the observation in Vlagtwedde/Vlaardingen alone but with stronger significance.

## Discussion

Our study is the first to show that one SNP in *TIMP1 *predicts excess FEV_1 _decline in two independent populations, albeit not quite reaching conventional statistical significance in the replication cohort. In the initial cohort we additionally found an association of *MMP2 *with FEV_1 _decline, but this was not replicated in the second independent population, indicating that the role of genetic variation in *MMP2 *on rate of FEV_1 _decline is still debatable. In contrast to previous reports on case-control studies that showed an association of *MMP1, MMP9 *and *MMP12 *with COPD, emphysema, decreased levels of FEV_1_, and/or excess decline in FEV_1 _[12,14]., we found no indication whatsoever for a role of *MMP1, MMP9 *or *MMP12 *in the development of (mild to moderate) COPD or FEV_1 _decline in our prospective population studies. Consequently, our data suggest that the imbalance in MMPs and TIMPs is likely not disturbed due to genetic variations in *MMP *genes. This does not rule out that MMPs play a role in COPD development at all. Genetic variations in genes involved in regulation of MMPs and TIMPs levels, such as interleukin(IL)-10, IL-13, epithelial growth factor (EGF) and tumor necrosis factor-α (TNF-α) [27,28]may clearly influence the imbalance of MMPs and TIMPs in COPD. Future studies are needed that address the effect of these genes on FEV_1 _decline in the general population.

We show for the first time that genetic variation in *TIMP1 *may accelerate the normally occurring FEV_1 _decline in the general population. We found that the common SNP Phe124Phe was associated with excess FEV_1 _decline in males only. Of importance, this association was replicated in the Doetinchem cohort with a larger genotype effect (-9.0 ml/yr and -4.2 ml/yr excess FEV_1 _decline in Doetinchem and Vlagtwedde/Vlaardingen respectively), but with somewhat lower significance (p values 0.10 and 0.04 respectively). Since the *TIMP1 *gene is located on the X-chromosome, carriage of one mutant allele may already account for an effect in males, whereas one mutant allele may be compensated by the other allele in females. However, another mechanism has to play a role since females homozygous for the mutant allele have a similar decline as heterozygous carriers.

Since the Phe124Phe SNP is a synonymous mutation and therefore unlikely having a functional effect on protein structure or function, it should be regarded as a marker for genetic variation in *TIMP1*. Future studies are warranted to identify SNPs that have a functional effect in this gene. Such SNPs may alter TIMP1 protein structure, resulting in an altered/diminished affinity for MMP9 and subsequently excess MMP9 activity leading to parenchymal destruction.

We observed opposite effects of the *TIMP1 *Ile158Ile SNP in the two populations under study. The SNP was associated with excess FEV_1 _decline in both females and males in Vlagtwedde/Vlaardingen, and with less FEV_1 _decline in females, without an effect in males in the Doetinchem cohort. The SNP has a very low prevalence and therefore type I errors can easily occur. By testing the SNP in an independent population, we can conclude that the significant effect in the Vlagtwedde/Vlaardingen population is possibly found by chance.

The *MMP2 *C-1306T genotype effect on FEV_1 _decline is small in the Vlagtwedde/Vlaardingen cohort, but we observed no effect at all in the Doetinchem cohort, which may indicate that the association in Vlagtwedde/Vlaardingen may possibly be a spurious result that is not relevant on a population level. On the other hand, we can not completely rule out a genetic effect of *MMP2 *since the power to detect small genotype effects on longitudinal lung function decline is much larger in the Vlagtwedde/Vlaardingen population due to the substantial longer follow-up time than in Doetinchem [29]. This may explain the lack of replication. Further studies with comparable power as in Vlagtwedde/Vlaardingen are warranted to elucidate the role of *MMP2 *in FEV_1 _decline in the general population.

*MMP9 *SNPs were not associated with development of COPD or FEV_1 _decline in our study. We did not genotype the *MMP9 *C-1562T SNP that was previously associated cross-sectionally with the presence of emphysema or COPD in Japanese and Chinese individuals in a case-control study [13,14]due to technical problems. Therefore, we cannot rule out a genetic role of *MMP9 *in COPD development. However, we tagged the whole MMP9 gene for haplotypes with a frequency above 10%, and found strong LD in the whole region. We are therefore confident that we also tagged the C-1562T SNP and that we did not miss information. Alternatively, the causative factor for higher levels of MMP9 in COPD lung tissue can be due to their transcriptional upregulation by other cytokines involved in COPD [30,31]It is therefore of interest to analyze SNPs in these genes in the future as well.

We did not confirm associations of the *MMP1 *and *MMP12 *SNPs and lung function decline as previously described by Joos *et al *and Hunninghake *et al *[11,12]However, in the first study the *MMP12 *Asn357Val SNP was only associated with rate of decline in FEV_1 _in combination with the *MMP1 *G-1607GG SNP. We performed the same type of analyses and found no association. Since Hunninghake *et al *found associations of *MMPs *and lung function in smokers, we also performed such stratified analyses according to smoking, but found no associations in the ever or current smokers.

Although the role of MMPs in COPD pathogenesis has clearly been demonstrated, we are the first to analyze the effects of SNPs in a cohort of the general population, whereas previous studies have used case-control designs. Moreover, differences in phenotypes make the comparison of our study and previous studies difficult. For example, several studies have investigated the effect of the C-1562T SNP in *MMP9 *in smokers and nonsmokers with respect to emphysematous phenotypes using chest CT scans [13,14,30]. We do not have CT scans available in Vlagtwedde/Vlaardingen or in Doetinchem, so we can not assess such genetic effects since pulmonary function tests are not very sensitive to detect (mild) emphysema [31].

Since the selection of our SNPs was hypothesis-driven, and we tested only 9 SNPs, we feel that a correction for multiple testing is not warranted, moreover since the strength of the current study lies in the replication of significant findings of one cohort in a second cohort. We feel we did not miss any clinically relevant associations of greater than 5 ml/year excess FEV_1 _decline due to lack of power. For example: we had approximately 1000 subjects with the wild type genotype of the rs8113877 in *MMP9 *and on average a mean annual decline in FEV_1 _of 17 ml/yr which results in a 80% power to detect an excess decline of 5.5 ml/yr in FEV_1 _in mutant carriers (n = 300), assuming a SD of 30 (derived from the actual SE = 1.166) in both groups. However, we may have missed associations of *MMP *SNPs with COPD, since we only had 40% power to detect an OR of 1.5, assuming a risk allele frequency of 0.25.

## Conclusions

Our study shows that genetic variation in *TIMP1 *is associated with excess FEV_1 _decline in two independent general populations, reaching moderate significance. Further research is needed to assess the functionality of this finding. We could not confirm a role for *MMP *SNPs in excess FEV_1 _decline and COPD development in the general population, although our study had sufficient power to detect genetic effects. Since SNPs in *MMP *do not appear to contribute to COPD, it is of interest to assess the genetic contribution of MMP modifying genes, like IL-10, IL-13, EGF, and TNF-α that regulate transcription of *MMPs*. In addition, SNPs in other *TIMPs*, such as *TIMP2*, may also affect the MMP-TIMP balance and thereby exert an effect on FEV_1 _decline in the general population.

## Competing interests

The authors declare that they have no competing interests.

## Authors' contributions

CCD performed the lab work, statistical analyses and drafted the manuscript. DSP is co principal investigator of the project, obtained funding of and supervised the project, and helped draft the manuscript. MS contributed to the statistical analyses. AB and HAS contributed to collection of the data. HMB is co principal investigator of the project, obtained funding of and supervised the project, and helped draft the manuscript. All authors read and approved the final manuscript.

## Supplementary Material

Additional file 1 **This manuscript contains an online supplement with additional methods**. click here for file
