# Supplementary material for: Genetic variation in TIMP1 but not MMPs predict excess FEV1 decline in two general population-based cohorts
Source: Respir Res. 2011 Apr 27;12(1):57. doi: 10.1186/1465-9921-12-57 (PMC3111362; doi:10.1186/1465-9921-12-57)
Supplement: Additional file 1 — This manuscript contains an online supplement with additional methods. [file 1465-9921-12-57-S1.DOC]

**Additional file to paper:**

**Genetic variation in *TIMP1* but not *MMPs* predict excess FEV_1_ decline in two general population-based cohorts**

CC van Diemen^1^, DS Postma^2^, M Siedlinski^1^, A Blokstra^3^, HA Smit^4^, and HM Boezen^1*^

Departments of ^1^Epidemiology and ^2^Pulmonology, University Medical Center Groningen, University of Groningen, Groningen, The Netherlands, ^3^ National Institute of Public Health and the Environment (RIVM), Bilthoven, ^4^Julius Center, University of Utrecht, The Netherlands

**Methods**

*Spirometry*

Pulmonary function measurements were performed with a water-sealed spirometer (Lode Spirograph, Lode Instruments, Groningen, The Netherlands). Measurement of inspiratory vital capacity (VC) after a deep expiration was followed by measurement of forced expiratory volume in 1 second (FEV_1_). The higher of the values obtained in two technically satisfactory tracings was taken as long as the difference between the two IVC values was less than 150 ml and the difference between two FEV_1_ values was less than 100 ml.

*DNA collection and Genotyping*

During the 1989/1990 survey of the Vlagtwedde/Vlaardingen cohort, neutrophil depot of spinned blood was collected and stored at -20°C. In 2003/2004 DNA was extracted from these samples with the QiaAmp® DNA Blood Mini Kit and checked for purity and concentration with the NanoDrop® ND-1000 UV-Vis Spectrophotometer (Nanodrop Technologies, Wilmington, Delaware, USA).

For all SNPs, primers and probes were obtained from Applied Biosystems TaqMan® SNP Genotyping Assays (Nieuwekerk aan de IJssel, The Netherlands), using the Assay-on demand service. Reactions were performed in 5 μl volumes and contained 10 ng DNA, 1x Taqman Universal Mastermix (Applied Biosystems), 200 nM of each probe and 900 nM of each primer. Cycling conditions on the ABI PRISM® 7900HT Sequence Detection System (Applied Biosystems) were 10 min 95ºC followed by 40 cycles of 15 seconds 95ºC and 1 minute 60ºC. End-point fluorescence was measured immediately after cycling. Alleles were assigned using SDS 2.1 software (Applied Biosystems). Genotyping reactions failed randomly with a maximum percentage of 6% and a minimum of 0%. There was no relationship between degree of missingness and evidence of association. We regenotyped 6% of the samples and found no errors in the genotypes, therefore our data are reliable and reproducible.

*Linear Mixed effect models*

We used a general genetic model, with wild types set as reference category, resulting in effect estimates of heterozygotes *vs.* wild types and homozygotes *vs.* wild types. Time was defined as time in years relative to the first FEV_1_, starting from the age of 30. Variables included in the model were sex, pack-years, the first FEV_1_ after age 30, and their interaction with time. Since including the level of the first FEV_1_ after age 30 and its interaction with time could introduce bias due to regression to the mean, these variables were also included in the model as random effect variables. The results of these analyses showed no change in estimates of the variables in the model or a better fit of the model, which indicates that there was no bias due to regression-to-the-mean. Therefore, the results are presented without these random effects
